# Neural system prediction and identification challenge

**DOI:** 10.3389/fninf.2013.00043

**Published:** 2013-12-25

**Authors:** Ioannis Vlachos, Yury V. Zaytsev, Sebastian Spreizer, Ad Aertsen, Arvind Kumar

**Affiliations:** ^1^Faculty of Biology, Bernstein Center Freiburg, University of FreiburgFreiburg im Breisgau, Germany; ^2^Simulation Laboratory Neuroscience – Bernstein Facility for Simulation and Database Technology, Institute for Advanced Simulation, Jülich Aachen Research Alliance, Jülich Research CenterJülich, Germany

**Keywords:** nuSPIC, spiking neural network, NEST, network function, network simulation

## Abstract

Can we infer the function of a biological neural network (BNN) if we know the connectivity and activity of all its constituent neurons?This question is at the core of neuroscience and, accordingly, various methods have been developed to record the activity and connectivity of as many neurons as possible. Surprisingly, there is no theoretical or computational demonstration that neuronal activity and connectivity are indeed sufficient to infer the function of a BNN. Therefore, we pose the Neural Systems Identification and Prediction Challenge (nu*SPIC*). We provide the connectivity and activity of all neurons and invite participants (1) to infer the functions implemented (hard-wired) in spiking neural networks (SNNs) by stimulating and recording the activity of neurons and, (2) to implement predefined mathematical/biological functions using SNNs. The nu*SPIC*s can be accessed via a web-interface to the NEST simulator and the user is not required to know any specific programming language. Furthermore, the nu*SPIC*s can be used as a teaching tool. Finally, nu*SPIC*s use the crowd-sourcing model to address scientific issues. With this computational approach we aim to identify which functions can be inferred by systematic recordings of neuronal activity and connectivity. In addition, nu*SPIC*s will help the design and application of new experimental paradigms based on the structure of the SNN and the presumed function which is to be discovered.

## Introduction

One strategy to approach the complexity of the brain is to adopt the three level description of Marr (1982) involving (1) identification of the problems that need to be solved by an animal to survive in its natural environment, i.e., *the computational level*, (2) identification of the algorithms delineating the solutions to the problems identified in the first step, i.e., *the algorithmic level*, and finally (3) investigation of how the available neuronal hardware is able to implement the algorithms identified in the second step, i.e., *the implementation level*. This scheme has successfully guided research in sensory information processing systems, particularly the visual system. However, for brain regions involved in higher-level computations such as in cognitive tasks, this approach has been less successful. An important, if not the main reason is that in these regions, which do not directly process information from the input/output stages (i.e., sensory and motor periphery), it is not easy to identify what problem the particular system might be solving. That is, there is often no plausible account of the *computational level* to begin with.

Nevertheless, in a somewhat modified form, David Marr's three level strategy is reflected in the search for the “neural correlates” of animal behavior. This approach has driven a multitude of experiments from which large amounts of data on task related behavioral observations together with concurrent neuronal activity recordings have accumulated. Yet, our understanding of brain function at the implementation level seldom goes beyond a mere *description* of such task related neuronal activity. Thus, in recent decades, computational and mathematical models have been designed to help to provide an *explanation* of the experimental data and, thereby, offer a “mechanistic understanding” of the neuronal processes possibly underlying sensori-motor and/or cognitive tasks. Unfortunately, though, most of these models have provided only limited insights thus far.

This lack of good mechanistic models is often attributed to the sparse sampling of the relevant neuronal activity. Indeed, even with the most advanced technology available only few dozens of neurons, mostly in close vicinity, can be reliably recorded over prolonged periods of time, whereas the behavior is likely to be governed by the activity of thousands or more neurons, distributed over multiple brain areas. Thus, there is a great interest among experimental neuroscientists to increase the numbers of simultaneously recorded neurons *in vivo* in behaving animals (Alivisatos et al., 2012). Progress on various innovative live-imaging methods as well as multi-electrode array recordings has indeed substantially increased the numbers of simultaneously recorded neurons. In parallel, there are attempts to fully map physical connections of the neurons in the brain area under investigation (Seung, 2009). The motivation underlying these large-scale projects is that the *full brain activity map* (Alivisatos et al., 2012) combined with the corresponding *connectome* (Seung, 2009) will substantially advance our understanding of neural computation in the brain at the *implementation level*.

Recent progress in the field of “connectomics” (Livet et al., 2007; Varshney et al., 2007; Hadjieconomou et al., 2011), together with bold claims on the potential benefit of mapping connectivity (Seung, 2012) and how it might help us understand even deeper problems like consciousness^1^ have sparked a huge public interest and support for initiatives such as the Human Connectome Project^2^ and the Human Brain Project^3^ in which a strong emphasis is put on reconstructing brain function from neuronal connectivity and biophysical properties extracted from multiple levels of organization. In some aspects, these human brain mapping initiatives are similar to the human genome project (Zador et al., 2012) and scientists have started to express concerns on allocating too much of our limited scientific resources to such projects^4^.

Here, with nu*SPIC*s our goal is to examine from a conceptual perspective whether there is indeed a scientific basis for the high hopes expressed by the proponents of these projects. Already Hopfield and Tank (1986) addressed this question and quite convincingly argued that the details of neuron and synapse properties, network connectivity and network activity are *insufficient* to extract the function of even a simple neuronal network. Indeed, it proved impossible to identify the function of a network composed only of seven neurons, despite complete knowledge of its connectivity and activity map (Hopfield and Tank, 1986). Unfortunately, with growing computer power and increasingly sophisticated experimental methods for mapping connectivity and sampling neuronal activity, it would seem that Hopfield and Tank's message has been forgotten.

In their study, Hopfield and colleagues focused on a specific class of networks (e.g., digital to analog conversion) and functions (e.g., recognition of spatio-temporal sequences) to highlight the limitations of the approaches at that time (Hopfield and Tank, 1986; Hopfield and Brody, 2000). Therefore, the question remains whether their conclusions also apply to current methods of investigating biological networks and their functions. To help test the generality of the arguments made by Hopfield and colleagues we introduce here the **N**e**u**ral **S**ystem **P**rediction and **I**dentification **C**hallenge (nu*SPIC*). The main goal of nu*SPIC* is to involve interested researchers from within and beyond the neuroscience community to determine which types of functions can be extracted by systematic experimentation on a given network and which experimental strategies yield the best possible results in such endeavor.

## The challenge

The **N**e**u**ral **S**ystem **P**rediction and **I**dentification **C**hallenge (nu*SPIC*) invites scientists, students and other interested individuals to:

1. Discover the function of a given spiking neural network (SNN) by performing a wide variety of experiments on a small network of spiking neurons (nu*SPIC* Class I).
  Each network is designed by the authors to implement one specific function (mathematical, logical, biological, etc.). The participant is provided with full information on the neuron properties and the connectivity matrix. This class of nu*SPIC* mimics our current experimental approach of recording the activity of neurons under different behavioral conditions in order to extract the function realized by the network.
2. Implement a given function using a finite number of neurons and synapses (nu*SPIC* Class II). This class of nu*SPIC* mimics our current approach of developing mechanistic models of task related neuronal activity recorded *in vivo*.

### Goals of the project

The main goal of neuroscience is to reverse engineer the function(s) of the brain, that is, to understand the mapping from stimulus to action. Some progress toward this goal has been made in the last decades in terms of associating certain basic operations with specific networks in the brain. For instance, it has been suggested that cortical circuits may implement a maximum function in combining visual features (Riesenhuber and Poggio, 1999). Similarly, canonical computations such as thresholding (Lo and Wang, 2006), linear filtering (Carandini et al., 2005), and normalization (Carandini and Heeger, 2012) have been proposed for various brain areas. There is also a strong support of the idea that computations in the brain are based on probabilistic principles (Doya, 2007). Moreover, neuroscience has indirectly benefited from theoretical progress in the field of machine learning, which has provided insights into the properties of feed-forward and recurrent neural networks. Indeed, it has been shown that feed-forward networks constitute a class of universal function approximators (Hornik et al., 1989) while recurrent networks are even more powerful, because they can be used to approximate any dynamical system (Funahashi and Nakamura, 1993). Despite the examples listed above, our understanding of *if* and *how* functions in general can be *extracted* by experimenting with neural networks is limited. Therefore, our aim with nu*SPIC* is to systematically examine how basic operations and more complex functions that are implemented in SNNs can be inferred by following an appropriate methodological approach. The specific goals are:

- To determine if it is possible to extract a function from a small SNN given full knowledge of neuronal activity and connectivity. Which classes of networks can be easily treated and which are more difficult or even impossible, in principle, to handle?
  To this end we will provide the participants with a variety of possibilities to interact and experiment with the SNN. In addition, the possibility of applying methods for “knowledge extraction” from neuronal networks similar to those used in the machine learning community (Tickle et al., 1998) will be considered.
- To document the specific strategies used by participants in extracting the function of a network and to use the successful strategies to improve experimental designs.
  To this end we will track the participants' experimentation histories and also interview successful participants on how they eventually reached the “correct” experiment to identify the network's function.
- To identify the technical and theoretical constraints in implementing different types of functions using SNNs.
  General rules of how logical/mathematical functions can be implemented do not exist. However, there are interesting approaches such as the one described in the neural engineering framework (NEF) by Eliasmith and Anderson (2003), which is based on population vectors and linear decoding. Alternative approaches, for instance based on attractor dynamics (Amit, 1989) or neuronal assemblies, have been proposed as well (Abeles, 1991; Bienenstock, 1995). Moreover, each strategy to implement a function may impose certain constraints on the neuronal hardware. For instance, NEF requires large numbers of neurons. Therefore, a dedicated effort to implement various types of functions in SNNs is a necessary step to understand the constraints of the chosen strategy on the design of functional neural circuits.

The goal of nu*SPIC* is neither to support nor abolish our current experimental paradigms in neuroscience. On the contrary, we would like to find out which classes of functions can be understood from the *full activity map* and the *connectome* of the underlying network. Thus, we hope to identify promising novel experimental paradigms, which are derived from the structure of the network and the complexity of the task.

### Target group

Neuroscientists, physicists, engineers, students, and other investigators with a genuine interest in understanding information processing in biological and artificial neuronal networks.

## Methods

Following the modern trends in application software development, we decided to implement nu*SPIC* as a web application, as opposed to a self-contained desktop software package. The basic prerequisites for using a web application are a reasonably modern web browser and a functioning Internet connection. This means that the participants do not need to install anything on their computer to use the software, and, in particular, that the nu*SPIC* could even be accessed using smartphones or tablets. It also has the advantage that we can easily support all major platforms such as Windows, Mac OS X and Linux at the same time and avoids the problem of having to deliver software updates. In addition, the background simulations can be run and processed on the server, which lowers the requirements on the performance of the participant's computer.

From an architectural point of view, nu*SPIC* features a highly modular design and consists of several building blocks that have become standard in state-of-the-art web application development: a front-end web server, a back-end application server, a web framework, a relational database and a queuing system. There are several popular, well-integrated stacks that provide this functionality, mainly differentiated by the language (Java, Python, Ruby) used as the universal glue between the stack components. We have chosen the Python stack as the foundation for nu*SPIC* mainly for two reasons:

First, most widely used neuronal network simulators (Gewaltig and Diesmann, 2007; Goodman and Brette, 2009; Hines et al., 2009) come with readily available Python interfaces, which renders it easy to build custom codes on top of these powerful tools. Likewise, another relevant package, PyNN (Davison et al., 2008), which provides a common interface that integrates many network simulators by enabling the creation of simulator-independent network descriptions to be simulated by any of the supported packages, is also a pure Python application.

Second, in recent years Python has become a de facto standard in neuroscientific simulations, data analysis and visualization, thanks to the aforementioned simulator bindings and an abundance of high-quality, ready-to-use scientific libraries (NumPy, SciPy, matplotlib, and others). Several online communities have formed around Python-based software in the field of neuroscience, such as NeuralEnsemble (http://neuralensemble.org), where, for instance, the aforementioned PyNN project is being developed, and NiPy, the “Neuroimaging in Python” effort (http://nipy.sourceforge.net).

Therefore, while we could have possibly built a similar system on top of Ruby on Rails, or a tool set from the Java ecosystem, the process of integrating neuroscience-specific code into such stack would have been unnecessarily complicated.

### nu*SPIC* architecture

The system is deployed on a virtual private server kindly provided by the German Neuroinformatics Node (G-Node), running Scientific Linux 6 (a freely available rebuild of Red Hat Enterprise Linux, backed by Fermilab/DoE and CERN). The system is managed through the Puppet configuration management software suite. The complete infrastructure description is hosted in a private git repository in the form of Puppet manifests and is fetched into the server when configuration updates need to be performed.

Puppet enables us to describe the configuration of the system in a declarative domain specific language (DSL) built on top of Ruby, which makes it possible to automatically re-create identically configured systems and serves as documentation at the same time. Additionally, it eases repetitive software maintenance tasks and allows to consistently enforce a particular state of the system (i.e., to monitor a set of running services and restart them in case of crashes, etc.).

We use GitHub as the development platform: the source code for the web application is released under the liberal terms of the MIT license and is publicly available at our GitHub organization profile (https://github.com/nuSPIC/). In addition, GitHub is hosting a number of private repositories for us, containing infrastructural package pools and Puppet manifests.

**Figure 1:**
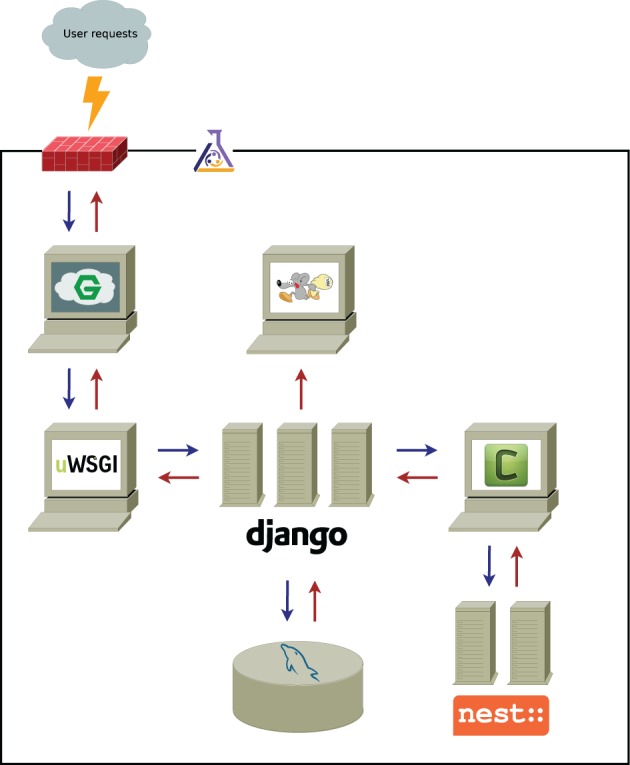
**Functional scheme of nu*SPIC* architecture**. Black solid line represents the LAN (local area network) boundaries and the gray cloud symbolizes WAN (wide-area network); the firewall is denoted by the red brick wall. The flask shows that all infrastructure delimited by the black line is managed by Puppet. Computers are a metaphor for individual services, and, in particular, servers stand for services, that spawn a number of independent workers to concurrently processes incoming requests. The computer with the running mouse icon represents Postfix, the mail server, the meaning of other icons is explained in the main text. Arrows show the flow of communications between the components of the system.

The overall scheme of the application is presented in Figure 1. User requests coming from the wide-area network (WAN) are filtered through the firewall and processed by the front-end web server. Requests for dynamic content (e.g., simulation data, as opposed to static content, such as images, JavaScript files, etc.) are passed on to the application server and processed by one of the Django workers.

We use nginx and uWSGI (depicted as computers with stylized “G” and “uWSGI” in Figure 1) as the front-end server (load balancer) and the back-end (application) server, respectively. Both are high performance, robust and low-footprint packages.

In the case that the request is computationally demanding and might take a significant amount of time to complete, it is pushed into the queuing system for background processing and the confirmation is immediately returned to the user. Queued requests (simulations) are picked up by task queue worker processes, executed, and the results are saved in the database.

As indicated in Figure 1, the nu*SPIC* application is built on top of the Django framework, that has been described as “the web framework for perfectionists with deadlines” (https://www.djangoproject.com). The reason for this choice is that it provides us with all necessary abstractions for rapid application development, such as an object-relational mapper (ORM), an automatically generated interface for site administrators and a powerful templating system. At the same time, it encourages clean model-view-controller (MVC) application design, while delivering uncompromised performance. We use mySQL as the relational database back-end for Django (depicted as a cylinder with a blue dolphin in Figure 1); it is widely considered as a default choice for this purpose and is powerful enough to handle our workloads.

The web interface (front-end) is developed using jQuery as the basic general purpose JavaScript library, in combination with a number of standard and custom plug-ins. We also use the D3 visualization library for plotting, as well as for graph layouts and vector graphics manipulations.

The task queue functionality is handled by Celery (indicated by a computer with letter “C” in Figure 1). It integrates well with Django and (conveniently) can use Django ORM as a back-end instead of a fully-fledged advanced message queuing protocol (AMQP) server. Since nu*SPIC* is a relatively low-traffic application, it is beneficial to have a solution that is easy to maintain for the performance that it provides, and yet have the possibility to upscale in a straightforward fashion, that is, to switch to a more robust high performance AMQP server, such as RabbitMQ, if needed.

The simulation code for the task processors was implemented in Python using the PyNEST API of the NEST simulator (Gewaltig and Diesmann, 2007), a neuronal network simulation software with emphasis on performance, correctness and reproducibility. We have chosen this simulator mainly for being well-experienced with its APIs. However, nu*SPIC* can be trivially extended to any other simulator that exposes a Python API that worker processes can make use of.

Table 1 summarizes the software packages used for various purposes in the nu*SPIC* project.

**Table 1:** **Software list and corresponding official websites, where it can be obtained**.

**Component**	**Name**	**Website**
Deployment platform	Scientific Linux 6	http://www.scientificlinux.org
Configuration management	Puppet	http://puppetlabs.com
Front-end web server	nginx	http://nginx.org
Back-end application server	uWSGI	http://github.com/unbit
Web application code	nu*SPIC*	http://github.com/nuspic
Relational database	mySQL	http://www.mysql.com
Web application framework	Django	http://www.djangoproject.com
General purpose JavaScript library	jQuery	http://jquery.com
Visualization in JavaScript	D3	http://d3js.org
Task queue implementation	Celery	http://celeryproject.org
Mail transfer agent (MTA)	Postfix	http://www.postfix.org
Neural network simulator	NEST	http://www.nest-initiative.org
Version control system	git	http://git-scm.com

## nu*SPIC*

### nu*SPIC* features

**Figure 2:**
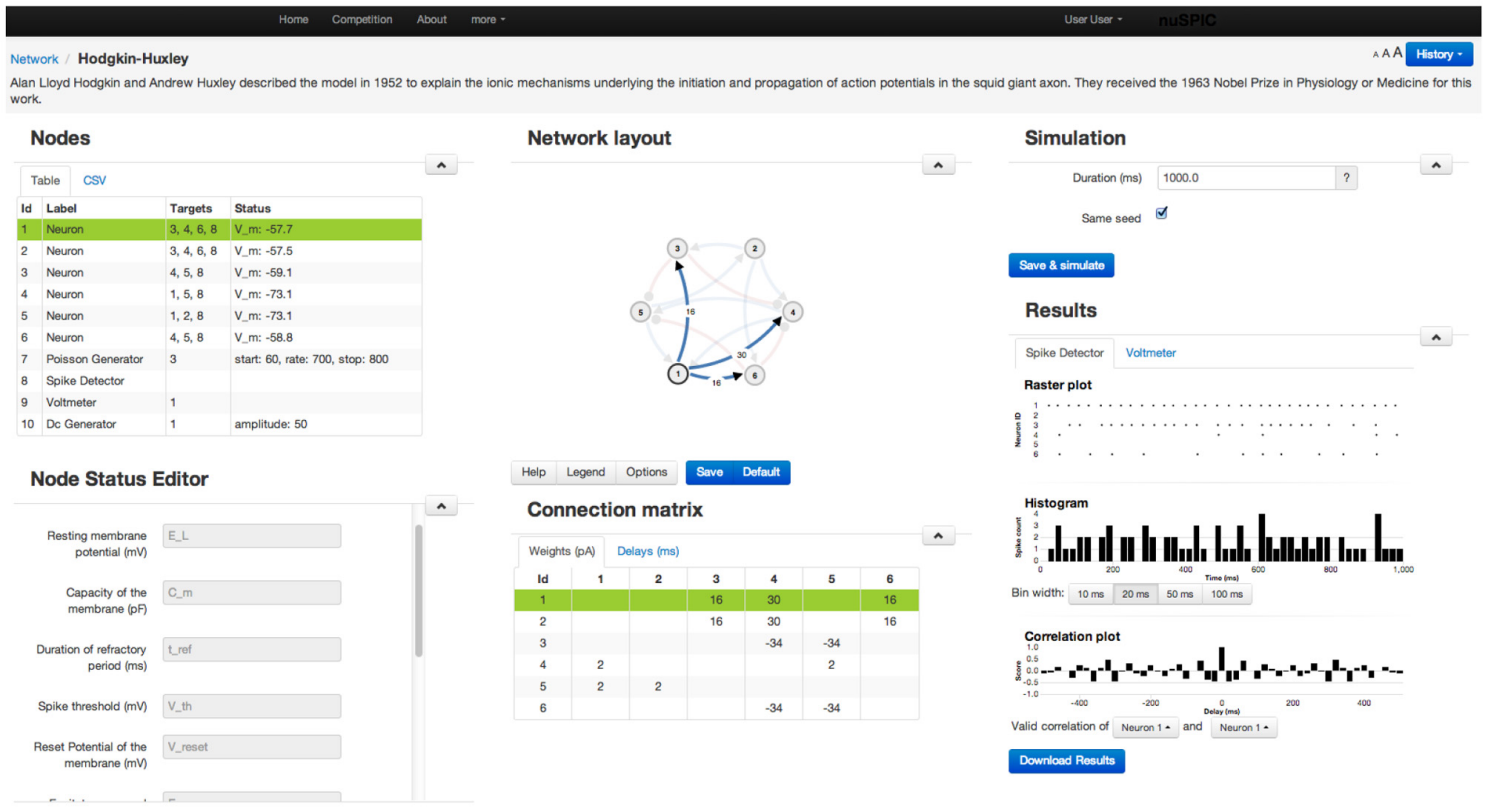
**A screenshot of the web-page showing the GUI for a Class I nu*SPIC* network**. Various parameters of the neurons, synapses, recording and stimulating devices can be easily changed using the appropriate menus. Similarly, users can view the output of the network in the form of the peri-stimulus time histogram, cross-correlogram of the spiking activity and sub-threshold membrane potential (cf. Figure 3). For further analysis the network output can be downloaded.

Because our target group goes beyond neuroscientists, we do not expect our users to be familiar with any specific neuronal network simulator or to have access to certain specific hardware to be able to perform nu*SPIC* simulations. Therefore, we designed a graphical user interface to enable users to perform simulations on a remote server (Figure 2).

**Figure 3:**
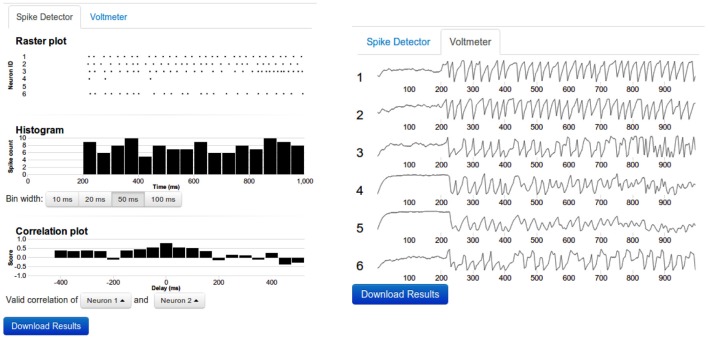
**Sample output**. **Left**: The top panel shows the raster diagram of spikes of every neuron that was connected to a spike detector device. The middle panel depicts the Peri Stimulus Time Histogram (PSTH) of the population activity. The bottom panel shows the cross-correlogram of the activity of two neurons. The bin width of the PSTH and cross-correlograms are same. By clicking on the diagram, the data can be viewed in more detail. **Right**: The voltmeter view contains stacked thumbnails of all neurons which are connected to a Voltmeter device. On mouse-over, the voltage and time appear for the dot in the corresponding voltage diagram. By clicking one of the voltage diagrams, a zoom-in view can be obtained. In such zoom-in view, it is possible to resize or to shift the zoom window.

- *Network:* The diagram of every nu*SPIC* network is provided once the particular circuit is loaded. Users can change the diagram and save it for later use. Neurons receiving external inputs are marked. Excitatory and inhibitory connections are drawn in different colors. Next to the network diagram, a connectivity matrix of the network is also provided. Both the network diagram and the connectivity matrix are updated whenever the user makes any changes.
- *Control of simulations:* The duration of the simulation and the type of input and output devices can be easily changed using appropriate menus. Using the Multi-Simulation View users can compare the output of two simulations with different parameter settings. It is also possible to add comments to each new simulation.
- *Input devices:* To facilitate probing of the network with different types of input we provide a flexible interface to choose from various devices, such as a Poisson spike generator, direct current injection, sinusoidal current input, spike generator (injects spikes into a neuron at specified times), and a noise generator all available in NEST. Any input device can be added, modified, reconnected, or deleted from an existing network. For each device a detailed explanation about its properties is provided. A neuron can receive multiple input devices. Once an input device is connected to a neuron, the network diagram is updated to include this input device.
- *Output devices:* Two output devices, Voltmeter and Spike Detector, are provided to record the membrane potential and the spiking activity of the neurons, respectively. The recorded membrane potential and the spiking activity are displayed after the simulation is completed (Figure 3). Both devices can be attached to multiple neurons simultaneously.
- *Data export:* Preliminary analysis of the data (such as peri-stimulus time histogram (PSTH), cross-correlations) can be performed on the web interface. However, it is not possible to envision all various analysis approaches a user may want apply. Therefore, the user has the option to download the results of the simulation of the network for off-line analysis. We also provide the source code of the web application to the users if they want to run the simulations offline, including the module that simulates network activity using NEST APIs.
- *Simulation history:* Once the simulation completes successfully, the data of the network and its results are stored on the server and the simulation history is updated. If the network simulation has been previously performed the user will be notified. Users are also able to reload previous versions of the network and the data.
- *Discussion forum:* A community page is provided to enable discussions among the users.
- *Submission of results:* The actual function of a network depends on the particular choice of time scales, kind of neural code, set of input and output neurons and decoding strategy etc. Therefore, we ask the users to send us an e-mail where such details associated with their specific experimentation can be described for further evaluation.

### Available classes of nu*SPIC*s

Currently we provide two types of nu*SPIC*s.

#### nu*SPIC* I

In the first class of nu*SPIC*s the goal is to discover the function of a small spiking neuronal network. To this end the user is supposed to perform a variety of experiments (in the spirit of experimental neuroscience) on small networks of spiking neurons, implemented as leaky-integrate-and-fire models and connected by current-based synapses.

Each of the networks has been designed to implement a specific function (e.g., a mathematical function, a logical operation, etc.). The task of the user is to extract all possible information about the function that is carried out by the network. A wide repertoire of stimulating and recording devices is provided; future versions of nu*SPIC* will include additional possibilities. Besides having flexible access to the networks, the user is also provided with full information on the network connectivity and the synapse and neuron parameters. More detailed information can be obtained at the corresponding web-page of nu*SPIC* (http://nuspic.g-node.org/).

#### Example of an implemented function

**Figure 4:**
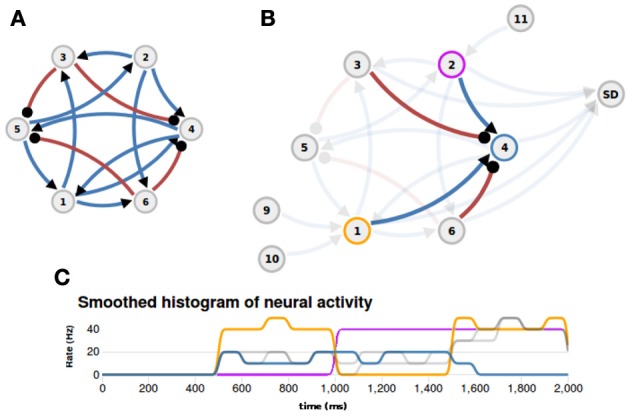
**Example (XOR function)**. **(A)** The layout of the network that implements a XOR function (blue: excitatory connections, red: inhibitory connections). **(B)** Appropriate inputs (500–1000 ms: Poisson input to N1; 1000–1500 ms: Poisson input to N2; 1500–2000 ms: Poisson input to N1 and N2) are provided through nodes N9, N10, and N11, and the spiking activity of neuron N4 is measured with a spike detector (SD). The afferent projections to neuron N4 are highlighted. **(C)** The histogram of neural activity reveals the XOR-function: the activity of ouput neuron N4 (blue line) is high only if one input neuron, N1 (orange line) or N2 (magenta line), is active and is zero otherwise.

To better illustrate the concept of nuSPIC we provide on the nu*SPIC* web-page a detailed explanation of how to proceed in order to extract the function implemented in a given network. In this example we “hardwired” the logical XOR-function, i.e., a two-input boolean function assuming the logical value “1” if and only if one of its inputs is “1” and otherwise assuming the value “0”. The network layout is shown in Figure 4A. It consists of six neurons (four excitatory and two inhibitory) that are recurrently connected by current based synapses. No information other than the connectivity, delays and weight matrices is provided. The user can probe the response of the network by stimulating individual neurons with various inputs and measuring the spiking activity and the membrane potential of putative output neurons. First, we need to identify which neurons are input nodes and which are output nodes. This is, in general, a cumbersome process, however, in this particular case, a more careful examination of the connectivity matrix already shows that neurons N1 and N2 are potential input candidates, because these neurons have only outgoing projections. Here, we are interested in the extraction of a boolean function, therefore, the interpretation of the virtual experiments must be made on the premise that the underlying neural code is binary, that is below (above) a threshold the activity of a given neuron has to be interpreted as a logical 0 (1).

Indeed, if neurons N1 and N2 are stimulated with an appropriate input sequence (e.g., 500–1000 ms: Poisson input to N1; 1000–1500 ms: Poisson input to N2; 1500–2000 ms: Poisson input to N1 and N2) and the spiking activity of neuron N4 is recorded (Figure 4B) then inspection of the firing rate response reveals that the output of neuron N4 could be interpreted as the logical XOR operation given the inputs to neurons N1 and N2 (cf. Figure 4C, and http://nuspic.g-node.org for more details).

#### nu*SPIC* II

Is it possible to implement *any*, physically realizable (Stannett, 2006), function using a network of spiking neurons? If yes, then how many neurons and synapses are needed and how should they be inter-connected? If not, is it then possible to delineate the types of functions that can be implemented from those that cannot? To address these questions, the second class of nu*SPIC*s is introduced. Here, the goal is to implement a function using a finite number of spiking neurons and synapses. The current version of nu*SPIC* only includes leaky-integrate-fire neurons and static current-based synapses. In subsequent versions we will introduce different types of dynamics, plastic synapses as well as neurons endowed with specific spiking behavior.

Our first challenge in this category is to implement a rate estimator^5^. With the development of the project more challenges will be added. It is also planned to enable users to suggest novel nu*SPIC*s themselves. We expect that the growing nu*SPIC* user community will eventually take the lead and more nu*SPIC*s will become available.

### Evaluation of submissions

One way of evaluating the users' submitted responses is to manually examine the results of their simulation. However, we are currently testing different methods for an automatic assessment procedure. For instance, the firing rates defined by the user in specific time windows could be compared with predefined target firing rates. Alternatively, the similarity between spike trains could be estimated (Victor, 2005). Criteria such as the choice of the relevant variables, that is, the choice of the “neural code”, the level of the signal-to-noise ratio (e.g., for spike-detection) etc. will ultimately need to be considered in the design of the evaluation methods.

### A powerful teaching AID

We realize that with a powerful, yet user-friendly web interface, nu*SPIC*s could become a useful teaching-aid to introduce spiking neuronal networks to students, starting already at high school level. Thus, we are currently implementing a third class of networks along with the associated nu*SPIC*s, where we will present textbook examples on the functioning of simple SNNs. This class of networks could also serve as a tutorial to proceed with the first two classes of nu*SPIC*s. The web interface will allow the user to alter various parameters to study the dynamics and behavior of the network and to create new networks in order to test certain hypotheses. Importantly, with nu*SPIC*s students do not need to learn any scientific computing language or syntax to operate the tutorials as would be the case for popular neuronal simulators such as NEURON, NEST, MOOSE. The simulator coming closest to nu*SPIC*s in this regard is “Nengo”, where a simple GUI is provided to design and run neuronal network simulations.

### Crowd sourcing

nu*SPIC* can be considered a scientific crowd sourcing project, falling in the category of a “citizen science” (Hand, 2010) or “networked science” (Nielsen, 2011) approach e.g., SETI@home (Anderson et al., 2002), Galaxy Zoo (Lintott et al., 2008), FoldIt (Cooper et al., 2010), Dream (http://www.the-dream-project.org/). This approach is also being used in two other initiatives in neuroscience: to devise a neuron model to predict experimentally measured spike trains (Gerstner and Naud, 2009) and to reconstruct neuronal morphologies (Gillette et al., 2011). Similarly, nu*SPIC* invites professional neuroscientists and scientists from other disciplines, as well as amateurs or laymen who are interested in dealing with problems that could provide insights into basic mechanisms of brain function. The underlying assumption is that individuals from different backgrounds have different knowledge, skills and viewpoints and thus, can contribute in a variety of ways that are not necessarily exploited by just one community alone. At first glance, nu*SPIC* is a brute force attack taking advantage of the huge number of people that can work on a problem in parallel. However, it goes beyond this by offering a platform to initiate and foster discussions among the users. This way, ideas can be exchanged, concepts for solutions can be developed in collaboration and successful strategies can be adopted from one problem to another. The hope is that eventually we will achieve a reasonable understanding of the types of functions that can be implemented with small spiking neuron networks and of the best possible methods to identify and extract those functions.

### Initial results

Before announcing the nu*SPIC*s to the public we had a trial run with the neuroscience community at the Bernstein Center Freiburg. This included mathematicians, engineers, physicists, and biologists, all highly experienced with both theoretical and experimental aspects of neuroscience. At this stage, the responses of the participants were evaluated manually. However, automatic procedures are currently being implemented (see section Evaluation of Submissions). A number of improvements were made to the nu*SPIC* design, based on already received feedback. We also made several interesting observations that have important conceptual implications:

- Despite being provided with complete knowledge of network connectivity and activity and despite having the possibility to probe and analyze the network with a variety of methods, almost all participants were unable to extract the function of any given network. Out of 20 participants, only one came close in identifying the function of one particular network composed of only seven neurons. This was achieved by close inspection of the connectivity matrix, without initially considering the network activity. However, the same strategy was not even remotely successful for other nu*SPIC*s. These results are suggestive of the difficulties that do arise when analyzing more realistic neural networks composed of a much larger number of neurons.
- In one case a function was identified that was not explicitly implemented. Indeed, under the limited set of “virtual” experiments performed on the network, its behavior could be interpreted in two different ways: one describing the actual function designed by the developers and a second, “emergent” function, not considered in the network design. This suggests that it may not always be possible even *in principle* to extract the function of a network in an unequivocal way. Moreover, it is important to point out, that all information extracted from a network may, by necessity, depend of the particular experimental method(s) used to probe and analyze the network.
- One of the test users wanted a way to silence one specific synapse of a neuron. However, manipulation of specific synapses was not implemented as a “virtual” experiment in nu*SPIC*s, because we knew that silencing a particular synapse in these networks would not provide any clues to extract the function. However, in real life experiments, when such information is not available, mechanisms to silence specific synapses of a neuron could be considered as an important potential clue and multiple research groups could spend years of research effort to develop a technique that could achieve this. Indeed, such a technique will generate a lot of experimental, presumably hypothesis driven data that will, however, not necessarily promote our original goal of extracting the function performed by the network. This clearly demonstrates that the lack of awareness of the potential relevance of certain network components or connectivity features for our main objective of identifying network function may severely impede progress. A theoretical or conceptual examination of the tasks at hand from the very onset could help to set the right priorities in our research agenda.

The results of this test trial revealed that in order to infer a function that is implemented by a given SNN the following parameters need to be specified: (1) set of input nodes, (2) set of output nodes, (3) neural code, (4) temporal scales, and (5) decoding method. In real experiments, the sets of input and output neurons can in some cases be constrained by including additional information on the afferents and efferents of the particular brain area that is being recorded from. To mimic this, we do provide information about the input and output nodes in some of the networks included in nu*SPIC* I. Note that a different set of nodes may lead to identification of a different function, that is, the identified function does depend on the chosen inputs and outputs. The results of the identification procedure will also depend on the assumed neural code underlying the local computations. Long lists of possible codes used by the brain, based on firing rates, temporal patterns, single neuron and population activity, have been proposed (Perkel and Bullock, 1968; Eggermont, 1998; Rieke et al., 1999; Decharms and Zador, 2000). In this competition we do not address the issue of neural coding explicitly. However, we do emphasize that choosing a particular coding scheme will affect the identification of the function. It is also important to point out that a network could be interpreted as implementing two or more coding schemes at the same time. It is then the role of the researcher to determine which scheme is more likely or meaningful in each particular case.

An additional parameter that determines the identified function is related to the temporal scale of the specified input and the recorded output signals. That is, neural responses at different timescales can encode different attributes of the input signals (Riehle, 1997; Panzeri et al., 2010). Thus, the exact time window, in which input and output signals are analyzed is crucial. Finally, a critical aspect for reverse engineering a function is the choice of the particular decoding method. Having identified input and output neurons and relevant variables (e.g. firing rates) and determined temporal windows for analysis, one still needs to apply a decoding algorithm. Several options exist, ranging from statistical methods (Knill and Pouget, 2004) to non-linear dynamics. Indeed, the particular choice will have an impact on the identification procedure. The above list of parameters is by no means complete, because additional factors such as choosing an appropriate model for noise, using continuous or discrete dynamics etc. will influence the task at hand.

It is evident that the concept of a function is contingent on a set of criteria that need to be defined prior to making any attempt of inferring the implementation of a particular function. This also illustrates that there is no unequivocal way to derive a function, because choosing a different set of criteria may lead to a different implementation. Thus, it is more meaningful to try to constrain the set of possible functions being implemented by a certain network, rather than trying to identify any single function. Therefore, the goal of nu*SPIC* is not necessarily to infer a specific function, instead, we intend to identify experimentally viable strategies by which a certain set of functions subject to the above criteria can be extracted.

## Discussion

Recently two new large-scale research projects have been launched. The first one is aiming at the construction of a full connectivity map of the brain. This is referred to as the *Connectome* project (Seung, 2009). The second one is an attempt to reconstruct also the full *activity* map emerging from the connectivity (Alivisatos et al., 2012) that gave rise to the recently announced BRAIN initiative (http://www.nih.gov/science/brain/index.htm). Both projects are based on the assumption that sufficient knowledge about neuronal connectivity and neuronal activity will eventually help us to develop a good understanding of neuronal network function as well. However, this assumption was seriously challenged already two decades ago by Hopfield and Tank (1986), and other researchers since then (Lazebnik, 2004; Marom et al., 2009; Kumar et al., 2013). Moreover, the machine learning community is well aware of the fact that it is not a trivial problem at all to understand *how* an artificial neural network, which is arguably much simpler than a biological neural network (BNN), solves a task that it has been trained for (Benitez et al., 1997; Tickle et al., 1998). With growing computer power, advancement of experimental methods that allow for increasing high-density sampling and modulation of neuronal activity and the success of data-driven approaches in other fields (e.g. data mining, genetic sequencing, etc.), it would seem that these concerns have been forgotten.

In an attempt to bring this issue back into the focus of the neuroscientific community, we have initiated the nu*SPIC* challenge. This challenge invites participants to identify the function of small spiking neuron networks by a series of realistic experimentations, akin to those performed by experimental neuroscientists. In addition, participants can design a spiking neural network (SNN) themselves to perform a specific function, a method often applied by theoretical neuroscientists when creating a computational model to explain empirical data.

Besides neuroscientists we also invite individuals (or teams) from other disciplines to participate. For this reason the challenge is run via a web-interface and a particular knowledge of operating neuronal network simulators is not required. This way we hope to tap into the high cognitive surplus of a huge pool of investigators.

The results of our first trial with a small group of neuroscientists at the Bernstein Center Freiburg suggest that our current conceptual tools and experimental methods do have severe limitations. In fact, it seems that their applicability is much more restricted than we would liked to admit. That is, in oversimplified neuronal networks—simple neuron models with linear dynamics and no spatial extent, simple static synapses with no plasticity, etc.—that theoreticians often prefer to study, operated under conditions that experimentalists would not dare to dream of—full and simple connectivity scheme, clear, stable, and unambiguous single-neuron recordings, no interference from other areas, no external noise sources, no experimental artifacts—it is, nevertheless, non-trivial to extract the function of a particular network. This only underscores how challenging it is to understand information processing in biological neuronal networks under “real”, *in vivo* conditions.

One could argue that biological neuronal networks implement different functions compared to those we used in our nu*SPIC* networks. Indeed, we do not claim that the functions we implemented in our model networks are necessarily biologically motivated. The point we want to make is that extracting even trivial functions such as those implemented in our “oversimplified networks” is not straightforward.

As stated earlier, the aim of nu*SPIC* is not to discard the current experimental and theoretical approaches. Neither do we intend to undermine the importance of experimental data for understanding the principle of neural information processing. On the contrary, with nu*SPIC*s we hope to learn, given a particular function and network connectivity, which data would be most informative to acquire. The success of nu*SPIC*s will help us draw important conclusions about the way certain types of networks should be best probed and analyzed in order to extract the implemented functions. Moreover, it will lead to a better understanding of the limitations of currently used methods. Thus, the success of nu*SPIC* will be determined by the novel experimental protocols and theoretical paradigms it will bring us.

### Conflict of interest statement

The authors declare that the research was conducted in the absence of any commercial or financial relationships that could be construed as a potential conflict of interest.

## Footnotes

^1^Interview with neuroscientist Jeff Lichtman, http://www.guardian.co.uk/science/video/2012/may/07/human-connectome-project-roadmap-brain-video?

^2^http://humanconnectome.org/

^3^http://www.humanbrainproject.eu/

^4^http://www.columbia.edu/event/neuwrite-presents-does-brains-wiring-make-us-who-we-are-57838.html, http://www.dradio.de/dlf/sendungen/wib/2071939/

^5^See http://nuspic.g-node.org/network/2/1/
